# Algorithm Change Protocols in the Regulation of Adaptive Machine Learning–Based Medical Devices

**DOI:** 10.2196/30545

**Published:** 2021-10-26

**Authors:** Stephen Gilbert, Matthew Fenech, Martin Hirsch, Shubhanan Upadhyay, Andrea Biasiucci, Johannes Starlinger

**Affiliations:** 1 Ada Health GmbH Berlin Germany; 2 Else Kröner-Fresenius Center for Digital Health Faculty of Medicine Carl Gustav Carus Technische Universität Dresden Dresden Germany; 3 Una Health GmbH Berlin Germany; 4 Institute for AI in Medicine University Hospital of Giessen and Marburg Marburg Germany; 5 Laboratory for Investigative Neurophysiology University Hospital Center and University of Lausanne Lausanne Switzerland; 6 confinis ag Sursee Switzerland; 7 Howto Health GmbH Berlin Germany

**Keywords:** artificial intelligence, machine learning, regulation, algorithm change protocol, healthcare, regulatory framework, health care

## Abstract

One of the greatest strengths of artificial intelligence (AI) and machine learning (ML) approaches in health care is that their performance can be continually improved based on updates from automated learning from data. However, health care ML models are currently essentially regulated under provisions that were developed for an earlier age of slowly updated medical devices—requiring major documentation reshape and revalidation with every major update of the model generated by the ML algorithm. This creates minor problems for models that will be retrained and updated only occasionally, but major problems for models that will learn from data in real time or near real time. Regulators have announced action plans for fundamental changes in regulatory approaches. In this Viewpoint, we examine the current regulatory frameworks and developments in this domain. The status quo and recent developments are reviewed, and we argue that these innovative approaches to health care need matching innovative approaches to regulation and that these approaches will bring benefits for patients. International perspectives from the World Health Organization, and the Food and Drug Administration’s proposed approach, based around oversight of tool developers’ quality management systems and defined algorithm change protocols, offer a much-needed paradigm shift, and strive for a balanced approach to enabling rapid improvements in health care through AI innovation while simultaneously ensuring patient safety. The draft European Union (EU) regulatory framework indicates similar approaches, but no detail has yet been provided on how algorithm change protocols will be implemented in the EU. We argue that detail must be provided, and we describe how this could be done in a manner that would allow the full benefits of AI/ML-based innovation for EU patients and health care systems to be realized.

## Introduction

Automated image analysis and segmentation [1], autonomous soft tissue suturing [2], and brain-machine interfaces [3]: these are technologies that until recently were only science fiction imaginings. They now all represent the state of the art in ML-based health care tools and all share one characteristic: all these systems are trained on patient data and can be quickly and automatically improved through retraining and on the basis of new patient data. ML is a subset of AI approaches, and this Viewpoint deals with the subset of ML applications that are classified as medical devices. The concept that an ML model will remain static over time is anathema to the concept of learning. Technologies are rapidly advancing to allow true real-time machine learning, and when the regulatory regimes allow it, ML-based health care tools have the potential to “learn” from new observations and continuous use and to retrain their models fully “on the job” [4,5].

Many ML-based health care tools are classified in the European Union (EU) and United States (and most other jurisdictions) as Software as a Medical Device (referred to in this Viewpoint as ML-based SaMD, with this term used to refer to technologies that learned from patient data sets and that will be further trained after being placed on the market). Learning and updating models pose a regulatory challenge, more so for ML-based SaMD that will learn from data in real time or near real time. Changes of this type pose a new regulatory challenge: the changes will often affect the fundamental clinical safety, clinical performance, and clinical benefit of the algorithm. Should they require full regulatory reassessment, a process that generally takes many months? Alternatively, can novel, faster, robust methods of quality oversight and approval be established? This Viewpoint compares the differing proposals put forward by the US and EU regulatory bodies for adapting the existing medical device frameworks to include consideration of learning ML-based SaMD. The crux of our argument is that highly proactive responses from regulators are required. The US Food and Drug Administration (FDA) and the EU have proposed strategies. The FDA approach is structured and comprehensive, while the EU approach overlaps the US approach but lacks detail on requirements and has not involved detailed stakeholder consultation.

There is evidence that more nuanced regulation of ML-based SaMD is being developed. A 2021 position paper of the American Medical Informatics Association recommended proactive regulatory approaches to bring improvement in clinical decision support (CDS) regulation including transparency standards, real-world performance (RWP) monitoring requirements, and improved postmarket surveillance (PMS) strategies [6]. A recent external validation of a widely implemented proprietary sepsis prediction model, the Epic Sepsis Model (ESM), used in hundreds of hospitals throughout the United States, found that it has poorly identified the onset of sepsis [7]. The authors concluded that the widespread adoption of this CDS, despite its poor performance, raised fundamental concerns about sepsis management. In our view, it also raises regulatory oversight and PMS concerns. The ESM is a penalized logistic regression model, included as part of a widely used electronic health record system, and it was developed and validated based on data from 405,000 patient encounters in three health systems between 2013 and 2015 [7]. Although only limited information is publicly available about the ESM, we recognize that it is not an example of an adaptive CDS (as defined in [6]); however, the ML approach used has the potential to be used in future adaptive CDS systems. Even as an example of a static CDS system, periodic updates based on PMS/RWP monitoring would form part of the lifecycle of this CDS, greatly increasing patient safety. Many of the considerations in this Viewpoint are applicable to this example.

The regulation of ML-based SaMD has been identified as one of the more substantial barriers to their clinical adoption [8]. Explorations of ML-based medical software in the United States found that many tools are not regulated by the FDA, that there is no FDA-maintained public record/database of approved ML-based SaMD, that many devices are approved through the 510(k)-clearance route (claim of substantial equivalence to an already-approved device), and where specific clinical evidence was provided for approval, this was exclusively from retrospective rather than prospective data [7,8]. Some of the medical applications of ML discussed in [7,8] were classified by the FDA as low risk or not classified as ML-based SaMD. For low-risk applications, existing regulatory frameworks may be sufficient. However, for the higher risk class devices discussed by [8,9], and also for adaptive ML-based SaMD and autonomous applications, there is a requirement for smarter regulation in the EU, United States, and worldwide, both to ensure patient safety and to remove a hurdle to adoption and advancement of the technologies [10-13].

## The Current Regulatory Framework for Learning ML-Based SaMD

ML-based health care tools with a role in individual patient diagnosis or therapy are currently regulated in the United States and EU as Software as a Medical Device. The regulation of medical devices has been included in US legislation since the 1938 Federal Food, Drug, and Cosmetic Act (FD&C Act), and more comprehensively in the 1976 Medical Device Amendments to the FD&C Act and subsequent updates [14]. In the EU, a legislative framework has been in place since the Medical Devices Directive 93/42/EEC and the Active Implantable Medical Devices Directive 90/385/EEC and was recently updated to the Medical Device Regulation (MDR) [15,16]. Both US and EU frameworks heavily rely on guidelines and harmonized norms, which define a set of standardized best practices for the development and deployment of medical devices. Neither of the current regulatory frameworks in their present form adequately considers the special properties of ML-based systems.

Historically, as hardware medical devices preceded software medical devices, the principles of a relatively static product (ie, following linear steps from initial concept to early and later stages of development, verification, validation, clinical testing, approval, and market release) were logical. As software in/as medical devices became established, the fundamental principles of medical device regulation were adopted largely unchanged to software. Allowance was provided for special properties of software; however, the level of detail in the legislation itself was low [15] and was instead provided through a set of international standards, including standards for the software life cycle [17], software usability testing, validation, and release and update. Initially, these processes were required to proceed in a linear “waterfall” cascade that had to be followed for software updates, release, and approval [17]. Later international guidance (see [18]) provided approaches for the development of SaMD within agile frameworks, the generally recognized optimal approach to software design [19], which is an iterative and incremental model of software development. Whether using waterfall or agile approaches, the international guidelines provide methodologies to adequately verify and validate SaMD software, a prerequisite for its safety and effectiveness.

## Limitations of Current Approaches

This raises the question, “does the current legislation provide a regulatory approval framework for ML-based SaMD?” Although burdensome, the current EU approach can be (and has been) adopted for ML-based SaMD (see [20]). SaMD manufacturers can optimize their software development processes to maximize their efficiency in this linear process, particularly for documenting the effects of model change on SaMD performance between updates (these are aspects of “change control,” a fundamental principle of medical device quality management systems). When applied to ML-based SaMD, software verification and validation are not in themselves sufficient, as they do not ensure that the ML model is safe; it could have safety problems related either to low-quality input data or to a poorly designed ML algorithm.

Manufacturers tend to relegate information about ML model updates to software development life cycle (SDLC) activities and postmarket clinical follow-up (PMCF). Although conventional PMCF and SDLC are highly valuable activities, provided they are executed in the correct environment and phase, they are generally inadequate to address ML problems. SDLC activities focus on software design controls that do not ensure clinically acceptable ML performance (ie, a software can be perfectly written and documented and yet the ML that it hosts can fail because of ML model problems). One reason for the inadequacy of current PMCF practices for ML model updates is that they typically generate data on a sufficient number of patients only months to years after changes are made to the SaMD. In addition, historically, the data quantity typically explored in PMCF approaches has been insufficient for the requirements of modern data-driven learning algorithms. As discussed later in this Viewpoint, PMCF can be adapted to allow for the rapid gathering of detailed data related to ML model updates. The adaptation of PMCF to this purpose and the definition of a systematic “protocol” for the implications of this data stream on device regulatory status are the foundation of proposed novel regulatory approaches.

## Proposed Solutions: The US FDA Action Plan

The issues of the appropriateness of the ML algorithm and input data and the safety of the derived ML model are tackled to a degree in recent standards, some of which are still under development (see for example ISO [International Organization for Standardization]/IEC [International Electrotechnical Commission] TR 24028 on trustworthiness in AI [21] and ISO/IEC DTR 24027 on bias in AI systems and AI-aided decision-making [22]) but have not yet been addressed in a joined-up fashion in legislation. However, these issues are addressed by novel and comprehensive proposals in the US FDA’s 2021 action plan [23], which effectively provides a roadmap for ML model validation. The FDA has conducted a structured consultation and has published a comprehensive action plan on regulatory approval strategies for adaptive ML-based SaMD [23,24], which has been accompanied by a high degree of engagement with the themes in the literature [25-27]. The action plan does not yet fully resolve the problems described in this Viewpoint, as the action plan is not complete or implemented. Nevertheless, the proactive and open approach of the FDA is commendable.

The action plan clearly recognizes that adaptive ML-based SaMD presents a challenge to traditional approaches: “The FDA’s traditional paradigm of medical device regulation was not designed for adaptive artificial intelligence and machine learning technologies” [23]. The FDA started the formal online consultation process in April 2019 [28]—with contributions guided by a well-conceived detailed consultation document—and conducted a public workshop in February 2020, followed by the publication of the action plan in January 2021 [23]. The consultation document and action plan are based on the FDA’s premarket programs, and took into consideration the International Medical Device Regulators Forum’s (IMDRF) medical device risk categorization principles [29], the benefit-risk framework, software modifications guidance, and the organization-based total product life cycle approach. Good machine learning practice (GMLP) principles will be used to ensure rigorous ML-based SaMD development. Algorithm changes will be transparently labeled for users, while methodologies for ensuring robustness and identification and elimination of bias will be incorporated. For each device, a two-component predetermined change control plan (PCCP) is envisioned. This will include a SaMD prespecification (SPS)—a predetermined change control plan setting out the scope of the permissible modifications—and an algorithm change protocol (ACP; note that it is the prediction model that changes, see Textbox 1), which sets out the methodology used in the ML-based SaMD to implement the defined changes within the scope of the SPS. The ACP is a step-by-step delineation of procedures to be followed so that the modification achieves its goals and the ML-based SaMD remains safe and effective. The action plan is notable for its strengths in harnessing the iterative improvement power of ML-based SaMD, while at the same time ensuring patient safety through continuous RWP monitoring. As a next step, the FDA will publish a complete draft guidance on the PCCP in 2021 [23].

**Definitions of machine learning algorithm software and models. These definitions are set out explicitly here as some of the regulatory discussion documents use machine learning terminology imprecisely.**

**Artificial intelligence/machine learning algorithm**

Machine learning algorithms are mathematical procedures that are implemented in code and are run on data to create an output machine learning model. Machine learning algorithms perform pattern recognition tasks to learn from data. For example, a machine learning algorithm could be trained on physician-labeled radiographs used to develop a machine learning model for tumor detection.

**Artificial intelligence/machine learning software**

Machine learning software is the code (ie, programming language) implementation of the machine learning algorithm. It is possible to implement a machine learning algorithm in many alternative ways or different programming languages.

**Artificial intelligence/machine learning model**

The machine learning model is created by running a machine learning algorithm on data. The machine learning model represents what was learned by a machine learning algorithm. The machine learning model consists of model data and a prediction algorithm, which can be regarded as an automatically created computer program. Once created, the machine learning model can be used for a specific task (eg, the machine learning model can be applied to unlabeled radiographs to locate possible tumors).

## Do the Innovative ACP-Based Approaches Adequately Ensure Safety?

The status quo that the FDA action plan will alter has the foundational principle that a medical device should be clearly defined, definitively tested, and meticulously documented before approval and then should effectively have unchanged clinical safety, performance, and benefit on the market, and this should be ensured through tight change control processes and postmarket surveillance. Any substantial change in clinical behavior would require reapproval. This framework has advantages in the simplicity of traceability and maintenance of safety oversight. A disadvantage is that this framework prohibits the rapid change of ML-based SaMD. The FDA action plan effectively proposes the same system, except that a boundary of change of the clinical behavior of the adaptive ML-based SaMD can be predefined, along with methods to oversee the degree of change and the resulting effects while on the market. If this is a paradigm shift, it is a small one—it effectively shifts the approval at the stage of readiness of a new product revision and the postmarket evaluation of change to a premarket comprehensive consideration of the changes that would be acceptable for the device.

By definition, changes that do not fall within the predefined risk-assessed thresholds are not allowed and require the normal processes of examination by the regulator before approval for the market. The FDA noted in the action plan that “stakeholders provided specific feedback about the elements that might be included in the SPS/ACP to support safety and effectiveness as the SaMD and its associated algorithm(s) change over time” and has reacted to this by promising detailed guidance on what should be included in an SPS and ACP to support the safety and effectiveness of ML-based SaMD algorithms [23]. It is the view of the authors that the overall principles set out in the FDA action plan (ie, predefining acceptable clinical safety, performance, and benefit on the market, and conducting RWP monitoring of these) represent an approach that is both rational and proportionate, and one that would ensure patient safety, provided the regulator is sufficiently involved in the oversight of RWP monitoring data and evaluation of this data in the context of the PCCP.

## The EU Artificial Intelligence Act

Following a European Commission white paper on AI in February 2020 and a subsequent public consultation [30,31], the European Commission published a draft Artificial Intelligence Act in April 2021 [32]. This draft legislation lays down harmonized rules for AI applications and it extends classical EU product conformity and CE marking concepts to all “high-risk” AI applications. The draft legislation is very similar to MDR in its core approaches, which are based around product intended use and postmarket monitoring systems. All use of AI in medical devices is defined as “high-risk,” and the draft legislation is designed to be compatible with MDR [16], to be overseen by the same Notified Bodies as MDR (although the detail on how oversight will operate remains to be established), and devices are to be covered by a single CE-mark representing conformity to both MDR and the Artificial Intelligence Act [16,32]. It is striking that there is no single mention of ML in MDR, its annexes, or its associated guidance (MDR [16] and Medical Device Coordination Group guidelines [33,34]), despite the fact these documents were released in 2017 or later. Essentially, the new draft Artificial Intelligence Act extends MDR [16], bringing it into the AI era.

The FDA approach had a clear and focused published proposal on ML-based SaMD regulation to frame the discussion for the public consultation. In contrast, the European Commission consultation on the 2020 white paper that preceded the draft legislation did not have an associated published proposal and was broad, bringing in all high-risk AI applications, not just health care. We studied the contributions to the consultation, and although there are some well-considered submissions, overall, there was little focused discussion on precisely how ML-based SaMD should be overseen in the EU. This lack of detail in proposals is also reflected in the draft legislation. For the first time in EU medical device legislation, the draft describes the concept of ACPs, but—unlike in the FDA action plan [23]—these are implied, rather than being specifically named or their requirements being set out in detail. Likewise, the draft legislation does not set out an analogue to the FDA’s PCCP approach, although again, the need for this is implied.

The critical clause in the draft legislation is as follows:

In line with the commonly established notion of substantial modification for products regulated by Union harmonisation legislation, it is appropriate that an AI system undergoes a new conformity assessment whenever a change occurs which may affect the compliance of the system with this Regulation or when the intended purpose of the system changes. In addition, as regards AI systems which continue to ‘learn’ after being placed on the market or put into service (i.e., they automatically adapt how functions are carried out), it is necessary to provide rules establishing that changes to the algorithm and its performance that have been pre-determined by the provider and assessed at the moment of the conformity assessment should not constitute a substantial modification.

The importance of postmarket performance monitoring is described, but no details are provided on special considerations for this in the context of ML-based SaMD: “all providers should have a post-market monitoring system in place. This system is also key to ensure that the possible risks emerging from AI systems which continue to ‘learn’ after being placed on the market or put into service can be more efficiently and timely addressed.”

## EU Regulatory Oversight: What Is Still Needed?

In September 2020, a thorough analysis of the EU legal requirements for ML-based SaMD [35] was carried out by the European medical devices trade association (European Coordination Committee of the Radiological, Electromedical and Healthcare IT Industry [COCIR]), who concluded that deployment is possible in a way that is consistent with MDR, but recommended that practical guidance should be made available, supported by the development of international standards. More specifically, the group recommends that the international standard describing software life cycle processes (IEC 62304) [17] should be updated, requiring manufacturers to define an ACP for adaptive ML-based SaMD. COCIR’s recommendation has been included in the text of the new “under review” edition of the standard [36]. We agree that the updating of standards is an important stepping-stone toward a clear framework, but changes can be long in the incubation. Simply updating standards documents may not bring the clarity required by EU Notified Bodies to allow them to make the judgment calls required to approve learning ML-based SaMD. The modifications to the IEC standard are also discrete, and do not guarantee cohesiveness of the EU regulatory framework. Moreover, updates in standards are generally conducted by a narrow group of domain experts; in the example above, the expertise group will largely consist of medical device software life cycle experts. As fully acknowledged by the FDA, consultation on the design of new regulatory frameworks for adaptive ML-based SaMD should also bring together experts on postmarket surveillance, RWP measurement, clinical evaluation, and labelling, as well as patient representatives. Although not specifically stated by the FDA, we argue that experts in real-time adaptive ML approaches, which are likely to be increasingly proposed for ML-based SaMD, should also be a key part of these discussions. The awaited EU guidance was not published with the draft Artificial Intelligence Act, and as such there will be a clear legal requirement for ACPs for adaptive ML-based SaMD, but without detail on how ACPs should be implemented.

There are several major implications for the EU if standardized procedures are not provided for premarket review of adaptive ML-based SaMD, for ACP, or for manufacturer oversight of systems on the market. Unclear or unspecified regulatory approaches required to fulfil requirements could lead to frameworks that are too burdensome to be worthwhile for manufacturers to deploy their technologies in a particular region. This may put patients there at considerable disadvantage, as they may not be able to access new diagnostic, therapeutic, or preventive modalities, or may only be able to access them after a significant delay. Unclear regulatory strategies could also significantly disadvantage the growth and prosperity of EU AI businesses. Lastly, as discussed in the general context of EU and US medical device harmonization and regulation in [6,37], unclear regulatory requirements are unlikely to function to ensure safety as they will likely lead to highly uneven regulatory oversight and enforcement.

It is unclear the degree to which the detailed EU approaches to adaptive ML-based SaMD will piggyback on the results of the already well-progressed consultative process undertaken by the US FDA. Other international approaches, such as those of the IMDRF and the joint WHO/International Telecommunication Union strategy for an independent standard evaluation framework for ML model benchmarking [38], could also provide input to an EU approach; however, concerted and prompt action is required on the part of the European Commission, to consult on and define EU-specific guidelines for providers to enable them to “establish[...] that changes to the algorithm and its performance that have been pre-determined.” The benefits of the US FDA approach have been discussed at length in this Viewpoint but as described in detail in a July 2020 viewpoint by Cohen et al [19], there are aspects of the US approach that cannot easily be translated to the EU. EU-specific solutions are required in three domains: (1) EU data protection considerations relating to the update problem, (2) the relatively less established system in the EU for RWP monitoring, and (3) EU differences in public perceptions and stated community values regarding the role of AI. Point 2 may be partly addressed through complaint and incident registration in the proposed EU database for stand-alone high-risk AI systems but this is not yet sufficiently defined in the draft legislation to determine this with certainty.

It is our view that the EU needs to provide specific guidelines for adaptive ML-based SaMD ACPs and RWP monitoring. Waiting for coordination to be achieved through alignment of international standards is an approach without a proven track record of success, and it is unclear whether international approaches alone are sufficient for the EU’s special circumstances. This should not be the basis for the development of the EU’s health care ML-based SaMD ecosystem, on which we depend to bring the benefits of health care AI to European society and its economy. What is needed is a clear standardized approach, similar to the now 2-year-old approach of the FDA, which sets clear procedures required for ML-based SaMD approval and postmarket provider and regulatory oversight. This could be achieved, with or without a focused public consultation, through published guidance from the EU Medical Devices Coordination Group, which should bring together the aspects addressed in the FDA action plan, the COCIR report, and the developing harmonized standards [23,35,36].

The EU approach to regulation of medical devices has faced criticism for lacking both harmonization and approaches for ensuring patient safety [37]. Although both of these aspects have been improved due to the MDR [16] through the introduction of greater transparency for patients and a central vigilance and PMS database (EUDAMED), the key underlying problems of market fragmentation and lack of clarity and harmonization still exist [37]. The main issue that this Viewpoint addresses is the potential for continued lack of standardized adoption of proactive oversight of health AI, and therefore the potential for the EU Artificial Intelligence Act [32] to fail in its objectives of harmonizing AI regulation and ensuring future-ready oversight and the safety of EU ML-based SaMD. We call for a detailed action plan and public consultation, echoing the FDA’s 2020 approach, that will allow manufacturers and other stakeholders in the medical community to inform the discussion on requirements and to definitively understand requirements as they relate to oversight, market surveillance, and checkpoints of safety and performance, reactive to ML-based SaMD adoption (ie, the details of ACPs and associated RWP monitoring and ACP mechanisms).

## Abbreviations

ACP: algorithm change protocol
AI: artificial intelligence
CDS: clinical decision support
COCIR: European Coordination Committee of the Radiological, Electromedical and Healthcare IT Industry
FDA: Food and Drug Administration (US)
GMLP: good machine learning practices
IEC: International Electrotechnical Commission
IMDRF: International Medical Device Regulators Forum
ISO: International Organization for Standardization
MDR: Medical Device Regulation
ML: machine learning
PCCP: predetermined change control plan
PMCF: postmarket clinical follow-up
PMS: postmarket surveillance
RWP: real-world performance
SaMD: Software as a Medical Device
SDLC: software development life cycle
SPS: SaMD prespecification
